# Grand multiparity: is it still a risk in pregnancy?

**DOI:** 10.1186/1471-2393-13-241

**Published:** 2013-12-23

**Authors:** Andrew H Mgaya, Siriel N Massawe, Hussein L Kidanto, Hans N Mgaya

**Affiliations:** 1 Department of Obstetrics and Gynaecology, Muhimbili National Hospital, PO Box 65000, Dar es Salaam, Tanzania; 2 Department of Obstetrics and Gynaecology, School of Medicine, Muhimbili University of Health and Allied Sciences, PO Box 65001, Dar es Salaam, Tanzania

**Keywords:** Grand multiparity, Pregnancy outcome, Diabetes mellitus, Hypertension, Malpresentation, Abruptio placentae, Placenta previa

## Abstract

**Background:**

The association of grand multiparity and poor pregnancy outcome has not been consistent for decades. Classifying grand multiparous women as a high-risk group without clear evidence of a consistent association with adverse outcomes can lead to socioeconomic burdens to the mother, family and health systems. We compared the maternal and perinatal complications among grand multiparous and other multiparous women in Dar es Salaam in Tanzania.

**Methods:**

A cross-sectional study was undertaken at Muhimbili National Hospital (MNH). A standard questionnaire enquired the following variables: demographic characteristics, antenatal profile and detected obstetric risk factors as well as maternal and neonatal risk factors. Predictors of adverse outcomes in relation to grand multiparous women were assessed at p = 0.05.

**Results:**

Grand multiparas had twice the likelihood of malpresentation and a threefold higher prevalence of meconium-stained liquor and placenta previa compared with lower-parity women even when adjusted for age. Neonates delivered by grand multiparous women (12.1%) were at three-time greater risk of a low Apgar score compared with lower-parity women (5.4%) (odds ratio (OR), 2.9; 95% confidence interval (CI), 1.5–5.0). Grand multiparity and low birth weight were independently associated with a low Apgar score (OR, 2.4; 95%, CI 1.4–4.2 for GM; OR, 4.2; 95% CI, 2.3–7.8) for low birth weight.

**Conclusion:**

Grand multiparity remains a risk in pregnancy and is associated with an increased prevalence of maternal and neonatal complications (malpresentation, meconium-stained liquor, placenta previa and a low Apgar score) compared with other multiparous women who delivered at Muhimbili National Hospital.

## Background

The term “grand multipara” was introduced in 1934 by Solomon, who called grand multiparas the “the dangerous multiparas” [1]. In general, the older literature defines “grand multiparity” (GM) as parity >7 [2,3]. More recent reports select a definition of GM to start from a parity of 5 because the threshold of risks of any obstetric complication, neonatal morbidity, and perinatal death increase markedly at parity ≥5 [4,5].

“Developed” countries have a low prevalence of GM (3–4% of all births) [6] as a result of unlimited access to not only contraceptives but also antenatal care, skillful medical practitioners and adequate facilities for safe delivery. Hence, high parity is not considered to be a risk factor for pregnancy-related complications [7-9]. Conversely, a high prevalence of GM has been reported in “developing” countries [10-12].

In Tanzania, guidelines set by the Maternal and Child Health section of the Ministry of Health and Social welfare consider GM to be an obstetric risk. Furthermore, high parity has been deemed a burden to the family and health systems [13]. The Tanzania Demographic Health Survey (TDHS) for 2005 revealed a total fertility rate (TFR) of 5.7, which is statistically at the same level as rates estimated by the TDHS in 1996 (5.8 births) and 1999 (5.6 births). These data implied that, on average, a Tanzanian woman will bear 6 children [14]. The unmet need for family planning is ≥20%. Moreover, a study completed in rural Tanzania revealed that ≤60% of health workers were unaware of the definition of GM [15].

Despite good coverage of healthcare in Tanzania (90% of the population is <10 km from a healthcare facility), provision of health services remains inadequate because of poor accessibility and lack of equipment within health facilities [16]. Priorities in the allocation of health-service resources based on disease burden and evidence-based medicine within the health sector includes the identification of women whose pregnancies are at increased risk of complications. Hence, the few medical resources that are available are allocated to those in the greatest need. Hindrance to appropriate distribution of healthcare resources to mothers and children include a lack of recent accurate data on the magnitude and factors that influence adverse maternal and neonatal outcomes.

High parity and reduced inter-pregnancy interval are reported to be risk factors for poor maternal and perinatal outcome. These factors together or independently may predispose the mother to anemia, diabetes mellitus (DM), hypertension, malpresentation, abruptio placentae, placenta previa, post-partum hemorrhage due the uterine atony, and uterine rupture [17-19]. Poor perinatal outcomes include low birth weight, prematurity and perinatal mortality [20-23]. GM has also been associated with previous loss of pregnancy such as intrauterine fetal death and perinatal death [24]. Absence of risk related to GM has been reported in some studies [7-9] and partly supported in others [25-28], which related GM to poverty, social deprivation, late booking at antenatal clinics, and pre-existing chronic illnesses (including chronic hypertension and DM). Advanced maternal age of grand multiparas has been reported to be an independent risk factor of gestational DM, ante-partum hemorrhage, fetal distress, prematurity, low birth weight, perinatal mortality and chromosomal congenital abnormalities (particularly Down syndrome) [29,30]. In this regard, consideration of the confounding effect of advanced age of the grand multiparas is pivotal when analyzing the maternal and neonatal outcome of GM. For that reason, it is important to note that some studies [31,32] have associated high parity with an elevated risk to the pregnancy without adjusting for age in the analysis.

In the absence of clear and consistent evidence of the association of GM with adverse pregnancy outcomes, classifying grand multiparas as a high-risk group could increase the cost burden to families and health systems as well as physical and psychological stress to the mother and family.

The present study intended to answer the following research question: “Is GM a risk factor for adverse pregnancy outcome?” Our null hypothesis was: “There is no difference in pregnancy outcome in grand multiparous women compared with low-parity women (parity = 2–4)” We wished to estimate and compare the specified maternal and perinatal complications among grand multiparas and other multiparous women delivered at Muhimbili National Hospital (MNH; Dar es Salaam, Tanzania) and identify their associated risk factors for poor maternal and perinatal outcome.

## Methods

### Setting and design of the study

This was a prospective cross-sectional study done at MNH. MNH is a National referral hospital which also serves as a teaching hospital for the Muhimbili University of Health and Allied Sciences (MUHAS). Dar es Salaam has, according to the 2002 census, a population of ≈2.5 million and an annual growth in population of 4.3% [23]. Referrals that are served at MNH come from municipal hospitals and health centers in Dar es Salaam as well as Bagamoyo and Kisarawe district hospitals in the neighboring coastal region for antenatal care, delivery or intensive care. Some women come as self-referrals (especially those living near the hospital) and others come as private clients under Intramural Private Practise Management (IPPM). Sixty-to-eighty percent of women who attend antenatal clinics and/or who undergo delivery at MNH are classified as low-risk pregnancies. Antenatal clinics at MNH provide health education on the: danger signs in pregnancy; delivery preparedness; care of the newborn; contraception; and sexual transmitted infections (including HIV/AIDS). MNH provides basic and comprehensive emergency obstetric care.

The mean rate of delivery per year is 9,000 deliveries with a daily rate of delivery of 10–30. Primigravida constitutes 40% of cases whereas grand multiparas comprise 16–17% of all deliveries (MNH Obstetric Database, unpublished report). The labor ward of MNH has a capacity of 38 delivery beds and IPPM contributes to ≈15% of all deliveries. The labor wards have equipment related to vacuum extraction, stitching, vaginal examination and delivery trays. Oxytocin is the main uterotonic agent used and is widely available in the labor ward. Prostaglandins such as misoprostol are used occasionally but acquired only from commercial pharmacies and not stocked in the hospital pharmacy.

The obstetric wards are attended by 35 obstetricians working with 25 obstetrics and gynecology residents, 2 registrars and ≈25 nurse midwives. The nurses and support staff work 8 h a day covering three shifts. The labor ward is managed by 5 nurse midwives and 2 attendants per shift. The Doctors-on-call Team comprises 1 specialist, 2 obstetric residents and 1 intern physician on 24-h call. There are two obstetric operating theatres located adjacent to the maternity block and a private labor ward (IPPM Annex).

Upon admission for delivery, a nurse midwife screens all women before entering the labor ward. A brief history (personal information, next of kin, antenatal history, obstetrics history) is taken and required information entered in the labor ward register. The on-call doctor reviews the partogram, and undertakes the initial and subsequent obstetric examination until delivery. After a normal vaginal delivery, mothers and babies are observed in hospital for 6–10 h. Babies delivered by cesarean section (CS) or those with a low Apgar score (<7) are admitted to the neonatal ward (one floor above the labor ward). The neonatal ward also admits sick babies from other nearby hospitals.

### Study population and sampling

The study population consisted of all multiparas (para ≥2) delivered at the hospital labor ward from 1 July 2007 to 31 December 2007. Inclusion criteria were consecutive recruitment of all multiparas who delivered a single neonate at a gestation age of ≥28 weeks. Multiparas who delivered twins and those who were seriously ill to the extent of not being able to communicate were excluded. Women who did not consent to join the study were also excluded. Post-delivery, all multiparas were identified daily from the delivery register, admission book on the postnatal ward, and report books in the general ward. They were then listed and assessed for eligibility. Written informed consent was obtained from all parturients who met the inclusion criteria. The response rate was 100%. All eligible multiparas were recruited prospectively and data obtained consecutively until the sample size was reached. The sample size was computed from Epi™ Info ver6 (Centers for Disease Control and Prevention, Atlanta, GA, USA). The Minimum required sample size was 1020 where grand multiparas were 255 and a lower parity group of 765 based on the power of the study (1-β) of 80% (confidence interval (CI), 95%). The estimated ratio of unexposed-to-exposed group was 3:1. The expected least frequency of disease in the unexposed group was estimated to be 2.0%.

### Data collection

Data collection was done for 6 months. The principal investigator and two research assistants collected data throughout the day as recruitment proceeded. Data were collected in the postnatal ward (where the eligible participant was admitted after delivery). The researchers reviewed the clinical notes (including the partogram) to extract information according to the variables of interest laid down by the standard questionnaire.

The standard questionnaire had three sections. The first section was demographic characteristics such as age and parity. The second section focused on obstetric risk factors such as hypertension and DM in the current pregnancy, previous preterm delivery, previous instrumental or CS, and a history of perinatal death. The third section recorded delivery outcomes and neonatal outcomes such as birth weight (g), prematurity (gestational age <37 weeks), congenital malformations, Apgar score and perinatal deaths.

For participants who had uncomplicated normal deliveries, the questionnaire was administered ≈3–4 h after delivery. For those who delivered by CS and those who were severely ill, data collection was done when they were fully awake and able to respond adequately to the questions. The questionnaire was pretested for 3 days to assess flow of inquiry and the comprehensiveness of variables of interest, as well as to evaluate the consistency of the measurability of participants’ responses. After pre-testing, data were entered and analyzed to judge the appropriateness of the questions. The research assistants were adequately trained on administration of the questionnaire.

### Definition of terms

“Primiparity” was considered to be parity of one delivery in a non-gravid woman. the “Nulliparity” was considered to be parity of zero deliveries in a non-gravid woman. “Multiparity” was defined as parity of ≥2 deliveries. “Delivery” was considered in pregnancies of ≥28 weeks of gestation. For the purpose of this study, GM was defined as parity of ≥5 with previous pregnancies of ≥28 weeks of gestation. “Low parity” was defined as parity of 2–4 deliveries with previous pregnancies of ≥28 weeks of gestation. “Parturient” referred to women who had delivered (or had already undergone labor).

“Perinatal death” was defined as stillbirth of ≥28 weeks of gestation and early neonatal death. “Intrauterine fetal death” was defined as fetal death of ≥28 weeks of gestation. “Low birth weight” was defined as birth weight of <2500 g. “Very low birth weight” was defined as a newborn weighing <1500 g. Macrosomia was defined as birth weight ≥4000 g. “Low Apgar score” was defined as an Apgar score <7 in the 5th minute after delivery.

### Data analyses

Data entry and cleaning was done by Epi Info™ ver6 and then transferred to SPSS ver13.0 (SPSS, Chicago, IL, USA) for statistical analyses. Data cleaning included amending information that was seen to be incomplete, suspected to be incorrect or inappropriately completed through cross-checking with the case notes and ward report logs and removal of typographic errors and duplicated information. The chi-square χ^2^ test was used in the analysis of categorical variables. The Student’s *t*-test was used to analyze continuous variables. p = 0.05 was considered significant. Predictors for adverse outcome in relation to grand multiparas were assessed using logistic regression analyses.

### Ethical considerations

Written informed written consent was requested and obtained from all participants before study recruitment. Participants were assured of complete voluntary participation and, whether or not they decided to participate, medical care would not be affected. All data were coded, questionnaires were identified by numbers, and privacy was maintained during data collection to achieve strict confidentiality. Ethical clearance was granted by the Research and Publications Committee of MUHAS. The study began when permission was granted by the relevant authorities and the Executive Director of MNH.

## Results

**Figure 1:**
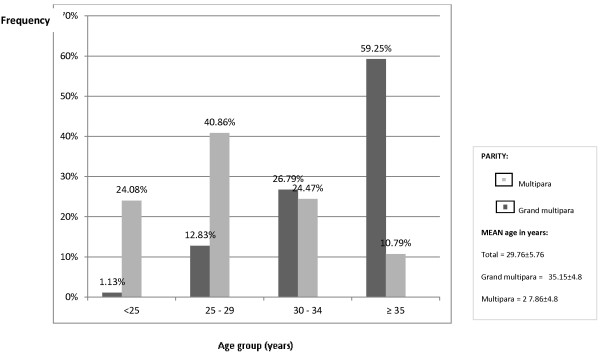
Percentage age distribution of the study population.

A total of 1809 multiparous women delivered during the study period. A total of 1025 multiparous women met the inclusion criteria and were assessed. The study group comprised 265 grand multiparas and 760 lower-parity parturients. The mean parity for the grand multiparas was 5.08 ± 1.64 whereas that of the lower-parity multiparas was 1.99 ± 1.2 (odds ratio (OR), 3.1; 95% CI, 2.9–3.2). The mean age among grand multiparas was 35.15 ± 4.8 years whereas that for other multiparas was 27.86 ± 5.7 years (OR, 7.2; 95% CI, 6.6–7.9). That is, grand multiparas were >8 years older than the lower-parity group (Figure 1).

Univariate analyses of antenatal profiles and obstetric risk factors (Table 1) showed grand multiparas to have a later booking for antenatal clinics (gestational age, 21.45 ± 5.9 weeks) compared with lower-parity women (19.49 ± 5.7 weeks) with an OR of 1.9 and 95% CI of 1.1–2.7. The prevalence of hypertension and DM in the current pregnancy was higher among grand multiparas but without significant differences when adjusted for age (p = 0.51, OR 1.6, 95% CI 0.3–9.8 and p = 0.06, OR 1.4, 95% CI 0.8–2.3, respectively). Obstetric history of preterm delivery and pregnancy as well as neonatal loss was significantly elevated (all p < 0.05) among grand multiparas. However, only history of previous preterm delivery (p < 0.001, OR 5.3; 95% CI, 3.1–8.9) and previous neonatal deaths (p < 0.001, OR 3.6; 95% CI 2.1–6.2) had persistent significant differences when adjusted for age.. Grand multiparas were more likely to deliver by CS (OR, 1.1; 95% CI, 0.6–1.7) and more closely associated with instrumental delivery (OR, 4.0; 95% CI, 0.5–29.1) although the difference was not significant at p = 0.79 and p = 0.61 respectively.

**Table 1:** Antenatal profile and obstetric risk factors according to parity in the study groups

**Variable**	**Grand multiparas**	**Multiparas**	**Unadjusted**	**Adjusted**
	**N = 265(%)**	**N =760(%)**	**OR (95% CI)**	**OR (95% CI)**
Gestational age at ANC booking	21.45 ± 5.9	29.49 ± 5.7	1.9 (1.1–2.7)	
Hypertension in current pregnancy	18.1	9.1	2.3 (0.6–8.6)	1.6 (0.28–9.8)
DM in current pregnancy	1.5	0.7	2.3 (0.5–3.4)	1.4 (0.8–2.3)
Previous abortion	26	16.2	1.6 (1.1–2.5)	1.2 (0.5–2.8)
Previous preterm delivery	35.1	5.8	8.8 (5.9–13.0)	5.3 (3.1–8.9)
Previous IUFD	21.5	7.4	3.4 (2.3–13.0)	1.2 (0.7–2.1)
Previous NND	27.2	4.9	7.3 (4.7–11.2)	3.6 (2.1–6.2)
Previous CS	16.6	20.5	0.7 (0.5–1.1)	1.1 (0.6–1.7)
Previous instrumental deliveries	1.5	0.7	2.3 (0.6–8.7)	4.0 (0.5–29.1)

Delivery outcomes (Table 2) showed that 62.1% of women had a spontaneous vaginal delivery, 37.3% had a surgical delivery and that 0.4% had a vacuum extraction. The mode of delivery did not differ significantly according to parity (all p > 0.69). Multiple regression analyses revealed that malpresentation; meconium-stained liquor and placenta previa were three-times more likely in grand multiparas than lower-parity women even when adjusted for age (all p ≤ 0.05).

**Table 2:** Maternal pregnancy outcomes according to parity in the study groups

**Variable**	**Grand multiparas**	**Multiparas**	**Unadjusted**	**Adjusted**
	**n = 265(%)**	**n = 760(%)**	**OR (95% CI)**	**OR (95% CI)**
**Mode of delivery**				
SVD	64.2	61.2	1.1 (0.8–1.5)	1
Surgical delivery	34.7	38.7	0.8 (0.6–1.1)	0.8 (0.6–1.1)
Vacuum extraction	1.1	0.1	8.6 (0.9–83.9)	8.2 (0.8–79.8)
**Maternal complications**				
Malpresentation	6.1	2.1	2.1 (1.1–4.1)	2.2 (1.1–5.0)
Premature delivery	12.5	10.5	1.1 (0.8–1.5)	0.8 (0.6–1.2)
Cord prolapse	0.4	0.9	0.4 (0.1–0.3)	0.6 (0.1–5.3)
Meconium-stained liquor	8.3	3	2.9 (1.6–5.3)	2.8 (1.3–5.9)
Abruptio placentae	2.6	1.7	1.5 (0.6–3.9)	1.8 (0.6–5.1)
Placenta previa	4.2	1.8	2.3 (1.0–5.1)	2.8 (1.1–7.1)
Uterine atony	4.8	1.4	3.2 (1.4–7.1)	2.0 (0.7–5.7)
Other	3	6.2	0.4 (0.2–1.0)	0.6 (0.2–1.4)

Table 3 displays neonatal outcomes according to parity. The mean birth weight was 3.003 ± 0.68 kg. Neonates delivered by grand multiparas were more closely associated with a low Apgar score (12.1%) compared with lower-parity women (5.4%) with an OR of 2.9 and a 95% CI of 1.5–5.0. Other factors associated with a low Apgar score were assessed by multivariate logistic regression (Table 4). GM and low birth weight were independently associated with a low Apgar score (p = 0.001, OR, 2.4; 95% CI, 1.4–4.2 for GM; p = 0.002, OR, 4.2; 95% CI, 2.3–7.8 for low birth weight).

**Table 3:** Neonatal outcome of the current pregnancy according to parity in the study groups

**Variable**	**Grand multiparas**	**Multiparas**	**Unadjusted**	**Adjusted**
	**N = 265(%)**	**N = 760(%)**	**OR (95% CI)**	**OR (95% CI)**
**Birth weight**				
Mean birth weight (kg)	3.08 ± 0.70	2.92 ± 0.67	1.2 (1.0–1.6)	1.2 (1.0–1.6)
Very low birth weight	1.9	3.3	0.5 (0.2–1.5)	0.8 (0.5–1.3)
Low birth weight	14.1	15.7	0.9 (0.6–1.3)	1.3 (0.8–2.3)
Normal birth weight	78	78.6	0.9 (0.6–1.2)	1
Macrosomia	6	3.4	1.9 (1.0–3.6)	0.8 (0.3–1.7)
**Stillbirths**	6.8	5.5	1.2 (0.7–2.2)	1.6 (0.8–3.3)
**Apgar score <7 (at 5th minute after delivery)**	12.1	5.4	2.1 (1.3–3.3)	2.9 (1.5–5.0)

**Table 4:** Logistic regression of risk factors correlated with a low Apgar score

**Variable**	**β**	**p-value**	**OR (95% CI)**
Grand multipara	0.88	0.002	2.4 (1.38–4.27)
Hypertension	0.04	0.89	1.0 (0.55–1.97)
Maternal age (>35 years)	-0.41	0.27	0.7 (0.31–1.45)
Low birth weight	1.45	0.001	4.2 (2.33–7.80)
Smoking	0.82	0.42	2.3 (0.29–17.25)
Alcohol intake	-0.602	0.23	0.5 (0.20–1.48)
Referrals	0.106	0.67	1.1 (0.67–1.83)

## Discussion

The present study showed a higher risk of maternal and neonatal complications such as malpresentation, meconium-stained liquor and placenta previa in grand multiparas as compared with lower-parity women even when adjusted for age. GM and low birth weight were independently associated with a low Apgar score. Advanced maternal age has been observed to carry an increased obstetric risk [33]. The age disparity between the two groups was managed by comparing the prevalence of important variables using age-adjusted ORs. A similar comparison methods has been used in other studies [23] whereas other authors have conducted age matching from the time of sampling [26].

We found the prevalence of hypertension and DM in pregnancy to be comparable between the two groups when age was adjusted. Similar studies have reached the same conclusion [3,7] but others [34-36] have found a significantly higher prevalence of hypertension and DM in grand multiparous women. A low prevalence of hypertension and DM among our participants could be attributed to a lack of statistical significance despite a twofold greater likelihood of grand multiparas having hypertension and DM than their lower-parity counterparts.

In the present study, the prevalence of a history of intrauterine fetal death was comparable between the two groups. However, a significantly higher prevalence of a history of preterm delivery and neonatal loss was evident in grand multiparas, a finding that was similar to a study conducted in Malaysia [23]. The reason for such findings could be related to a recurrence of pregnancy risk of fetal and neonatal death. Conversely, the association of a history of increased pregnancy loss with high parity could also be influenced by the need of the mother with previous fetal or neonatal loss to compensate for such a loss by attempting a successful pregnancy.

Statistical comparability with regard to the prevalence of vacuum-extraction delivery despite vacuum delivery being ten-times more frequent in grand multiparous women than lower-parity multiparous women was thought to be attributed to the infrequent availability of an appropriately functioning vacuum extractor at MNH during the time of the study. However, a high prevalence of CS (35–40%) cannot go unnoticed. This is because such a high prevalence of CS compared with a low prevalence of vacuum-extraction deliveries have been linked to an evolving tendency of obstetricians to avoid difficult deliveries such as those using vacuum extraction [37,38]. Lack of data on the use of vacuum extraction in low-resource settings has also been shown to hinder recommendations for the procedure [39]. An increasing prevalence of surgical deliveries seen in developed countries from the 1980s onwards is currently being realized in developing countries, including those in Sub-Saharan Africa [40-42]. An increased prevalence of CS in developing countries has not been shown to improve pregnancy outcomes [43] and, in some cases, has been shown to be associated with poor pregnancy outcomes [44].

There was a high prevalence of meconium-stained liquor, malpresentation and placenta previa in grand multiparas as compared with the lower-parity group, a finding that is in concurrence with other studies [4,21,45]. Meconium-stained liquor has been used as an indicator for fetal distress but it is a controversial marker for fetal compromise [46]. The passage of meconium can be a physiological response of a mature gastrointestinal tract of the fetus or relaxation of the anal sphincter in response to fetal hypoxia. Conclusive evidence of fetal distress is more closely related to the characteristics of variability in the fetal heart rate and acidemia [47,48]. Most of the malpresentations were of the breech type. In the absence of other obstetric indications for CS (e.g., footling, previous scarring, cord prolapse or prematurity) grand multiparas have a better performance in breech delivery than lower-parity women [49]. Therefore, a breech presentation may not necessarily be an added obstetric risk to grand multiparous women. Other maternal complications (premature labor, cord prolapse, abruptio placentae, uterine atony) were comparable between the two groups, in agreement with other studies [3,7,50]. The reason behind the comparability of uterine atony could be the practice of active management of the third stage of labor and the wide availability of uterotonic agents to all women delivered at the labor wards of the MNH.

As shown in previous studies [4], neonates born with low Apgar score were more closely associated with grand multiparas. In this study the independent predictors closely correlated with a low Apgar score was grandmultiparity and low birth weight. Hypertension, smoking, alcohol intake in pregnancy, or a mother being a referral case from another hospital with a risk of second- and third-level delay in receiving healthcare were not associated with a neonatal low Apgar score.

Despite a history of fetal or neonatal loss being a recurrent risk factor [4], grand multiparas in the present study showed a higher prevalence of a history of previous neonatal deaths rather than fetal and neonatal demise in the current pregnancy. Such a tendency could be associated with the impact of socioeconomic deprivation usually associated with grand multiparas. This state of deprivation leads to poor care of the newborn in the early or later neonatal period, thereby resulting in morbidity or mortality of the neonate [25], rather than the obstetric performance in the current pregnancy (which is more closely related to the health of the newborn).

Lack of an account of other confounders which affect the pregnancy outcome (e.g., inter-pregnancy interval, nutritional status, psychosocial status of the woman) was a limitation of the present study. Because of its cross-sectional design, the present study failed to make causal inferences of some risk factors (though it showed the prevalence of adverse pregnancy outcomes between grand multiparas and their lower-parity counterparts). Importantly, because of the low prevalence of some variables (e.g., vacuum-extraction deliveries, DM in pregnancy, cord prolapse, uterine atony), a bias in the comparability of events could have been present. Caution is required in translation of these institutional-study results based on outcome measures to the general population.

## Conclusion

This study demonstrated that GM remains a risk in pregnancy and is associated with an increased prevalence of maternal and neonatal complications (malpresentation, meconium-stained liquor, placenta previa and a low Apgar score) as compared with other multiparous women who delivered at MNH. From these findings, we recommend that:

1. The Ministry of Health and Social Welfare review the relevance of GM being considered a risk factor by searching for evidence through a population-based, nationwide study.

2. In low health-resource settings all pregnancies are prone to adverse outcomes, so adequate management of labor, a good referral system as well as the practice of basic and comprehensive obstetric emergency care should be mandatory.

## Competing interest

The authors declare that they have no competing interests.

## Authors’ contributions

AHM participated in design of the study, carried out the collection and analyses of data, and drafted the first and final version of the manuscript. SNM participated in the design of the study, was involved in the analyses, and drafted the first version of the manuscript. HLK participated in data analyses and helped in the development of the final version of the manuscript. HNM contributed in the design of the study and participated in the development of the first and final version of the manuscript. All authors read and approved the final manuscript.

## Authors’ information

**Dr. Andrew H. Mgaya MD, MMED;** Specialist Obstetrician Gynecologist, Muhimbili National Hospital.

**Professor Siriel N. Massawe MD, MMED, PhD;** Consultant Obstetrician Gynecologist, Muhimbili University of Health and Allied Sciences.

**Dr. Hussein L. Kidanto MD, MMED, PhD;** Consultant Obstetrician Gynecologist, Muhimbili National Hospital and honorary Senior Lecturer, Muhimbili University of Health and Allied Sciences.

**Professor Hans N. Mgaya, MD, MMED;** Consultant Obstetrician Gynaecologist, Muhimbili University of Health and Allied Sciences.

## Pre-publication history

The pre-publication history for this paper can be accessed here:

http://www.biomedcentral.com/1471-2393/13/241/prepub
